# Using Deep Learning Models of Gene Regulation to Guide Drug Prioritization

**DOI:** 10.3390/ph19071097

**Published:** 2026-07-16

**Authors:** Xiaoqin Huang, Ivan Ovcharenko

**Affiliations:** Division of Intramural Research, National Library of Medicine, National Institutes of Health, Bethesda, MD 20892, USA

**Keywords:** drug repurposing, noncoding variants, deep learning, transcriptomics, breast cancer

## Abstract

**Background:** Drug repurposing offers a cost-effective strategy to accelerate therapeutic discovery, but most computational approaches do not model noncoding genetic variation. Because over 90% of genome-wide association study (GWAS) risk variants reside in noncoding regions, linking regulatory variation to therapeutic hypotheses remains a major challenge. **Methods:** We developed an integrative deep learning framework that links allele-specific enhancer prediction to candidate therapeutics through two complementary prioritization strategies, a transcription factor (TF)-based and a gene-based approach. We used MCF7-breast cancer context as a proof-of-concept system. **Results:** GWAS heritability was significantly enriched in MCF7 enhancers. Allele-specific variant scoring identified 1537 breast cancer risk variants with strong predicted regulatory effects, and attribution-based motif discovery revealed enrichment of FOXA1-associated motif features, consistent with FOXA1 upregulation in primary tumors. TF-based prioritization, integrating FOXA1 knockdown-induced and drug-induced gene expression profiles, identified 63 candidate compounds, including 18 approved drugs, and recovered fulvestrant, an established breast cancer therapy. Gene-based prioritization, mapping candidate regulatory variants to 347 target genes, identified 140 candidate compounds, including approved breast cancer drugs toremifene and raloxifene. Both strategies identified compounds with anti-correlated transcriptional signatures across core breast cancer hallmark pathways, and integration of pathway anti-correlation, drug-gene interactions, and supporting experimental or clinical evidence yielded 15 high-confidence repurposing candidates. **Conclusions:** Recovery of approved breast cancer therapeutics supports the biological relevance of deep learning-predicted regulatory variants. This study establishes a regulatory variant-guided drug repurposing framework that connects noncoding genetic variation to candidate therapeutics and provides a scalable strategy for generating pharmacologically relevant hypotheses from the noncoding genome.

## 1. Introduction

The development of new therapeutics remains slow, costly, and characterized by high attrition rates [1,2,3,4,5]. Traditional drug discovery typically requires 10–17 years and costs over $2 billion per approved drug, with only approximately 11% of compounds entering clinical trials ultimately receiving approval, and success rates even lower in areas such as neurodegenerative diseases [6]. Drug repurposing offers a complementary strategy by identifying new indications for compounds with established safety profiles, thereby potentially accelerating therapeutic discovery while reducing cost and risk [7,8]. A growing number of computational approaches have been developed to facilitate drug repurposing, including gene expression signature matching, network-based inference, and structure-based modeling [1,9]. Many of these strategies leverage molecular phenotypes, such as drug-induced or disease-associated transcriptional profiles, to identify compounds with opposing or corrective effects, and have yielded promising results across diverse disease contexts, such as muscle atrophy [10], neurodegenerative disorders [11,12], and infectious diseases [13]. However, most existing frameworks rely primarily on transcriptional anti-correlation or network similarity and lack explicit modeling of how genetic risk perturbs disease-relevant regulatory programs.

Genome-wide association studies (GWAS) have revealed that over 90% of risk variants for complex traits reside in noncoding regions of the genome, particularly within distal regulatory elements such as enhancers [14,15,16]. Enhancers orchestrate cell type-specific transcriptional programs through cooperative binding of TFs, and perturbation of enhancer activity represents a major mechanism by which genetic variation influences disease risk [17]. However, most GWAS-informed drug discovery approaches prioritize genes based on genomic proximity or statistical association without explicitly modeling allele-specific regulatory effects within enhancers or the TF-associated gene expression changes through which these variants influence disease risk [18,19]. Consequently, how noncoding genetic risk variants can be translated into therapeutic hypotheses through their regulatory effects on gene expression remains poorly defined.

Recent advances in deep learning-based regulatory modeling enable prediction of enhancer activity directly from DNA sequence and inference of allele-specific effects of noncoding variants [20]. Among available regulatory sequence models, we adopted TREDNet because the central task of the present study is allele-specific enhancer variant-effect prediction rather than general regulatory annotation. Although models such as DeepSEA, Enformer, and Borzoi have been widely used for predicting chromatin features or gene-regulatory outputs from DNA sequence, previous comparative analyses from our group showed that TREDNet achieved stronger performance for enhancer variant-effect prediction across multiple datasets, despite modest advantages of some alternative models in enhancer classification tasks [21]. TREDNet has also been applied successfully in prior studies to prioritize candidate causal regulatory variants in multiple disease contexts [22,23,24], supporting its suitability for linking noncoding GWAS variants to enhancer-level regulatory effects. This modeling strategy provides a framework for moving beyond static association signals toward identification of TF-centered gene expression changes associated with genetic risk [21,22,25,26,27]. We therefore hypothesize that disease-associated noncoding variants disproportionately affect enhancer motif features associated with specific TFs, and that compounds that modulate the transcriptional output of these variants, either by targeting genes directly linked to variant-proximal regulatory regions or by modulating the gene expression regulated by the variant-implicated TFs, represent computationally supported candidates for therapeutic repurposing.

Here, we present an integrative framework that moves from noncoding genetic risk to therapeutic prioritization through two complementary strategies. The first links allele-specific enhancer predictions to candidate therapeutics by matching drug-induced responses to TF knockdown-induced transcriptional profiles. The second maps model-predicted putative regulatory variants to regulatory target genes and tests for drug induced differentially expressed gene enrichment in this gene set, providing independent path of variant-linked therapeutics. Our approach combines cell type-specific deep learning-based enhancer prediction, allele-specific variant impact assessment, and motif discovery to identify trait-relevant TFs, integration of TF knockdown-induced gene expression with drug-induced transcriptional responses to quantify directional concordance; variant-to-gene mapping for TF-independent gene-based enrichment under drug treatment; and pathway-level anti-correlation and curated drug-gene interactions to refine candidate compounds. We illustrate this strategy using breast cancer as a proof-of-concept application, demonstrating how risk-associated enhancer motif features preferentially converge on FOXA1-associated motifs and how compounds prioritized through both TF-based and gene-based strategies show anti-correlated transcriptional effects to core breast cancer-associated hallmark pathways, with successful recovery of approved breast cancer therapeutics providing supporting evidence for the biological plausibility of both approaches. This proof-of-concept application demonstrates a regulatory variant-guided drug repurposing framework that can be extended to other complex traits as matched epigenomic, TF perturbation, and drug transcriptional datasets become available.

## 2. Results

### 2.1. Overview of the Framework

**Figure 1:**
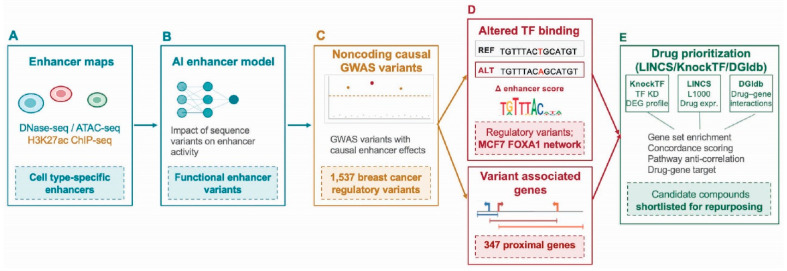
Regulatory variant-guided drug repurposing framework. (**A**) Cell type-specific enhancer definition from chromatin accessibility and H3K27ac profiles. (**B**) Cell type-specific enhancer models are trained using TREDNet. (**C**) Identification of candidate regulatory variants through allele-specific enhancer prediction. (**D**) Inference of TF-associated motifs using attribution-based motif discovery, and mapping of regulatory variants to 347 proximal genes, defined as the two nearest genes within 1000 kb either upstream or downstream of each variant. (**E**) Two complementary drug prioritization strategies: TF-based prioritization integrating FOXA1 knockdown profiles (KnockTF) with drug-induced gene expression (LINCS L1000), and gene-based prioritization through gene set enrichment in variant mapped genes; both refined by pathway anti-correlation and drug-gene interaction data (DGIdb) to yield candidate compounds shortlisted for repurposing.

The overall analytical framework is summarized in Figure 1. We first defined cell type-specific enhancers by integrating chromatin accessibility (ATAC-seq or DNase-seq) and H3K27ac profiles across multiple cell lines obtained from ENCODE [28]. Cell type-specific enhancer models were trained using TREDNet, a two-phase deep learning framework [22]. Phase one was pre-trained on 4560 genomic and epigenomic profiles from ENCODE v4 [29], and phase two models were fine-tuned for each cell type using 2-kb enhancer and control sequences (see Section 4). Using this model, GWAS variants were evaluated for allele-dependent differences in enhancer prediction, and candidate regulatory variants were identified based on allele-specific enhancer predictions. Attribution-based motif discovery (DeepLIFT [30] followed by TF-MoDISco [31]) was then applied to infer TF motif features preferentially associated with risk alleles.

To connect these regulatory variants to candidate therapeutics, we pursued two complementary drug prioritization strategies. The first, TF-based prioritization, integrates TF knockdown-induced transcriptional profiles with drug-induced gene expression from the LINCS L1000 dataset [32], prioritizing compounds based on transcriptional concordance with TF perturbation profiles from KnockTF 2.0 [33]. The second, gene-based prioritization, directly maps model-predicted putative regulatory variants to their proximal genes and tests for enrichment of drug-induced differentially expressed genes in this variant-linked gene set, providing a complementary approach independent of TF perturbation data. Both strategies are followed by pathway-level anti-correlation analysis and integration of drug-gene interaction data from DGIdb 5.0 [34] to refine candidate therapeutics.

### 2.2. Deep Learning Enhancer Modeling and Heritability Enrichment Link Cell Lines to Disease Gwas

Cell-specific enhancer models were trained using the TREDNet two-phase deep learning framework [22], achieving robust performance on chromosome-level held-out test sets (see Section 4), with AUROC of 0.91 and AUPRC of 0.65 in MCF7 cell line (Figure S1A,B). These results indicate reliable discrimination of enhancers from human open chromatin regions not overlapping enhancer sites, using a 1:10 positive-to-negative sampling ratio.

Significant heritability enrichment was observed for trait-cell line pairs consistent with known cell type identity. MCF7 enhancer annotations span 2.4% of HapMap3 SNPs yet account for 25.1% of breast cancer SNP heritability (10.5-fold enrichment; *p* = 4.1 × 10^−6^; one-sided z-test with block jackknife standard error; linkage disequilibrium score regression (LDSC) partitioned heritability regression [35]; Figure S1C), indicating that breast cancer genetic risk is disproportionately concentrated within MCF7-specific regulatory elements, consistent with MCF7’s origin as a breast cancer-derived cell line [36]. Based on this enrichment and the availability of matched TF perturbation and drug transcriptional data, we selected the MCF7-breast cancer pair for subsequent allele-specific modeling, TF inference, and drug prioritization analyses, as a proof-of-principle model.

### 2.3. Allele-Specific TF Convergence at Breast Cancer Risk Loci

Focusing on the MCF7 breast cancer context, 20,619 breast cancer-associated variants passing the genome-wide significance threshold (*p* < 5 × 10^−8^) from full GWAS summary statistics [37] were used. FUMA mapped these variants to 151 genomic risk loci. To summarize their broader genomic distribution, we also intersected them with a predefined GRCh38 European LD block map “https://github.com/jmacdon/LDblocks_GRCh38/blob/master/data/pyrho_EUR_LD_blocks.bed (accessed on 10 April 2026)”, which assigned 18,800 variants to 141 LD blocks, with many variants falling within the same block. Of the 20,619 significant variants, 830 variants overlapped MCF7 enhancers (1-kb windows). For each variant, we computed an allele-specific enhancer score difference (Δscore), defined as the difference in TREDNet-predicted enhancer probability between the risk and protective allele sequences centered on a 2-kb window (Δscore = P*_risk allele_* − P*_protective allele_*; see Section 4). Variants were retained as candidate regulatory variants if at least one allele exceeded the model-derived enhancer probability threshold (false positive rate < 10%) [23] and |Δscore| ranked within the top 10th percentile of the empirical distribution across all evaluated variants. This filtering identified 1537 variants (7.5%) exhibiting strong allele-dependent differences in predicted enhancer probability, with risk alleles associated with both increases and decreases in enhancer prediction score, consistent with the heterogeneous regulatory architecture of complex trait loci (Figure S2C). To provide independent functional support for these prioritized variants, we evaluated their enrichment in external functional genomics datasets. Candidate regulatory variants were significantly enriched for breast cancer eQTL [38] compared with all evaluated background variants (33 candidate overlaps among 1537 variants vs. 195 overlaps among 20,619 variants; odds ratio = 2.56, Fisher’s exact test *p* = 7.50 × 10^−6^; Table S1). In addition, candidate variants were strongly enriched in experimentally defined MCF7 FOXA1 ChIP-seq peaks (41 candidate overlaps vs. 145 overlaps among all evaluated variants; odds ratio = 5.00, Fisher’s exact test *p* = 3.91 × 10^−14^; Table S1). These results provide functional support that the prioritized variants are enriched for experimentally supported regulatory features related to gene-expression regulation and FOXA1 binding in the relevant cellular context.

To evaluate the robustness of the |Δscore| threshold, we performed a sensitivity analysis using alternative cutoffs. Candidate variants defined using the top 5%, 10%, 15%, 20%, and 25% |Δscore| thresholds were all significantly enriched for breast cancer eQTLs compared with background variants (Supplementary Table S2). The selected top 10% threshold showed the strongest enrichment (1537 variants; 33 eQTL overlaps; OR = 2.563; Fisher’s exact test *p* = 7.50 × 10^−6^), supporting this cutoff as a reasonable balance between functional enrichment and retaining sufficient candidate sequences for downstream motif discovery.

To identify TF motifs preferentially contributing to breast cancer risk allele-associated enhancer prediction, we applied attribution-based motif discovery to the 1537 candidate regulatory variants. DeepLIFT [30], a feature attribution method that estimates the contribution of each input base to a neural network prediction relative to a reference input, was used to compute nucleotide-resolution contribution scores from TREDNet predictions for each allele sequence. These contribution scores were then analyzed with TF-MoDISco [31], which identifies short high-contribution subsequences (“seqlets”) and clusters recurrent seqlets into TF motif classes. In this framework, DeepLIFT identifies nucleotide positions contributing to allelic differences in enhancer prediction, and TF-MoDISco summarizes these signals into interpretable TF-associated motif patterns. Across the 1537 candidate variants, this approach identified 32 TF motif classes with varying degrees of allelic enrichment in risk versus protective allele attribution profiles (Figure S2A; Table S3). Broadly acting architectural factors, including CTCF and SP2, showed no significant allelic bias (enrichment fold = 1.0 and 1.13, respectively), indicating comparable contributions to enhancer prediction in both allele contexts. In contrast, a subset of lineage-associated TFs showed preferential enrichment of motif signatures in risk allele attribution profiles. Most prominently, FOXA1-associated motif features were strongly enriched in risk alleles relative to protective alleles (40 vs. 0 seqlets in risk vs. protective alleles, *p* = 4.9 × 10^−12^), indicating that FOXA1 motif patterns contribute more strongly to TREDNet-predicted enhancer prediction in risk allele sequences. Related forkhead family members, including FOXD2, showed comparable directional enrichment (35 vs. 0 seqlets in risk vs. protective alleles). These results indicate that breast cancer risk alleles are disproportionately associated with forkhead family TF motifs.

We integrated model-derived allelic TF motif enrichment with tumor gene expression from TCGA-BRCA to further prioritize TFs. Of the 32 TF motif classes evaluated, 15 showed preferential enrichment in risk allele attribution profiles and 11 in protective allele profiles (Table S3). We first applied two data-driven criteria: (i) significant enrichment of TF motifs in risk versus protective allele attribution profiles, and (ii) concordant upregulation of the corresponding TF in primary breast tumors relative to matched normal tissue. Among TFs satisfying both criteria, FOXA1 and FOXD2 exhibited strong motif enrichment (40 vs. 0 and 35 vs. 0 seqlets in risk versus protective alleles, respectively) and high tumor-specific upregulation (log_2_ fold change (log_2_FC) = 2.18 and 2.26, respectively; *p* = 5.9 × 10^−167^ and 1.1 × 10^−69^, Table S3). In contrast, other TFs with highly enriched motifs, such as RXRG and BATF3, showed reduced expression in tumors (log_2_FC = −3.03 and −0.31, respectively; *p* = 9.8 × 10^−80^ and 1.9 × 10^−3^), indicating discordance between motif enrichment and transcriptional activity (Table S3). We next examined whether these prioritized TFs have reported roles in breast cancer regulatory programs. FOXA1 has been shown to bind enhancer regions and promote estrogen receptor recruitment in breast cancer cell models, thereby influencing estrogen receptor-dependent transcription [39,40,41]. Experimental modulation of FOXA1 in breast cancer models disrupts estrogen receptor-dependent transcription, alters enhancer accessibility, and reduces tumor cell proliferation [39,40]. In contrast, little evidence currently links FOXD2 to breast cancer-related regulatory processes. To assess the disease relevance of FOXA1-regulated transcriptional programs, we evaluated overlap between genes differentially expressed upon FOXA1 knockdown in MCF7 cells and genes dysregulated in TCGA-BRCA tumors. FOXA1 perturbation affected the expression of 1780 genes (false discovery rate (FDR) adjusted *p* < 0.05 from differential expression analysis, as reported in KnockTF 2.0, a curated database of TF knockdown and knockout gene expression profiles comprising 456 TFs across 357 human cell lines [33]), of which 205 (11.5%) overlapped with tumor-associated differentially expressed genes (odds ratio = 1.18; *p* = 0.024, one-sided Fisher’s exact test). Additionally, 202 FOXA1-regulated genes overlapped with genes mapped to breast cancer GWAS loci (FUMA [42]), supporting the relevance of FOXA1-regulated transcriptional programs to inherited disease risk. We therefore next focused drug prioritization on identifying compounds whose transcriptional effects are concordant with FOXA1 knockdown-induced gene expression changes.

Although multiple TF-associated motif classes were enriched in the allele-specific attribution profiles, downstream TF-based drug prioritization required compatible TF perturbation signatures from KnockTF. FOXA1 was therefore selected for the main downstream analysis because it was strongly enriched in the motif analysis and had a corresponding perturbation dataset suitable for transcriptional concordance and pathway-level analyses. Other enriched motifs were not carried forward because compatible perturbation data were unavailable or insufficient within the current framework.

### 2.4. Drug Candidate Prioritization Through Foxa1-Centered Transcriptional Concordance

To identify such compounds, we integrated the FOXA1 knockdown-induced gene expression profile from the MCF7 cell line (1780 differentially expressed genes; FDR adjusted *p* < 0.05 from differential expression analysis, as reported in KnockTF 2.0) with drug-induced transcriptional profiles from the LINCS L1000 dataset. For each compound in LINCS L1000, we computed a FOXA1 knockdown concordance score, defined as the mean drug-induced z-score of FOXA1 knockdown-upregulated genes minus the mean drug-induced z-score of FOXA1 knockdown-downregulated genes, such that positive values indicate transcriptional concordance with FOXA1 knockdown (see Section 4). Candidate compounds were required to satisfy two criteria: (i) statistically significant enrichment between drug-induced gene expression changes and FOXA1 knockdown-associated differentially expressed gene sets, and (ii) a FOXA1 knockdown concordance score within the top 10th percentile across all evaluated compounds. Approved drug enrichment was maximal and statistically significant exclusively at the 90th percentile threshold (2.1-fold; odds ratio = 2.5; *p* = 0.002, one-sided Fisher’s exact test), declining and losing significance at more stringent or more lenient thresholds (Table S4). This filtering yielded 63 candidate compounds (Table S5), of which 18 are approved for human clinical use by the FDA or equivalent regulatory agencies according to DrugCentral [43] (a curated database of international drug approval status) and ChEMBL [44] (a curated database of bioactive compounds with clinical development phase annotations), representing a 2.1-fold enrichment relative to the full LINCS L1000 library (odds ratio = 2.5; *p* = 0.002, one-sided Fisher’s exact test). To stratify candidates by clinical evidence, compounds were categorized into three tiers: Tier 1-approved drugs with registered breast cancer clinical trials in ClinicalTrials.gov [45]; Tier 2-approved drugs without breast cancer-specific trials; and Tier 3-investigational compounds (with or without breast cancer clinical trials).

**Figure 2:**
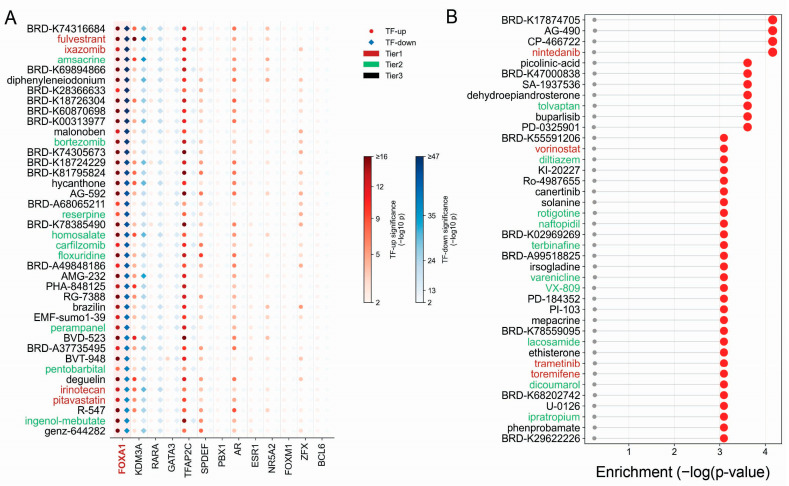
Top 40 compounds prioritized based on FOXA1-centered and variant-to-gene approach. (**A**) Directional transcriptional concordance between prioritized compounds and FOXA1 knockdown-induced gene expression. Heatmaps display the top 40 compounds ranked by FOXA1 knockdown concordance score whose drug-induced gene expression changes show directional concordance with FOXA1 knockdown-induced gene expression in MCF7. The x-axis shows all available MCF7 TF knockdown profiles (genes with FDR adjusted *p* < 0.05 from differential expression analysis, as reported in KnockTF 2.0); the y-axis shows compound names colored by candidate tier. Dot color and intensity represent enrichment significance (−log_10_ *p*-value, one-sided Fisher’s exact test); with darker colors indicating greater significance. Red circles indicate enrichment among TF knockdown-induced upregulated genes, whereas blue diamonds indicate enrichment among TF knockdown-induced downregulated genes. (**B**) Enrichment of candidate variants mapped genes in compound-induced down-regulated genes compared with random gene sets in prioritized compounds. For each compound signature, enrichment of candidate genes in the top 250 down-regulated genes was evaluated using Fisher’s exact test. The top 40 signatures are shown ranked by candidate-gene enrichment significance. Red points indicate −log_10_ *p*-value from the candidate gene set, and gray points indicate mean −log_10_ *p*-value from random gene sets matched for gene-set size.

Notably, our model has solidly recovered fulvestrant, an FDA approved drug for treating metastatic breast cancer in postmenopausal women [46]. To characterize transcriptional patterns among the 63 FOXA1-prioritized compounds, we computed enrichment of all available MCF7 TF knockdown gene sets from KnockTF 2.0 against drug-induced gene expression profiles. For each TF independently, we quantified directional concordance by measuring whether genes upregulated upon TF knockdown were also upregulated by the drug (up-up) and whether genes downregulated upon TF knockdown were also downregulated by the drug (down-down) (Figure 2A). This concordance framework was used because our goal was to identify compounds whose transcriptional effects mirror the expression pattern induced by TF knockdown, such that higher concordance indicates greater similarity between the drug-induced profile and the TF knockdown signature. Although compounds were selected based on FOXA1 concordance, multiple additional TF-associated gene sets showed consistent enrichment across prioritized compounds, indicating that these drugs may influence coordinated transcriptional programs beyond FOXA1 alone. Among TFs with significant directional concordant enrichment, TFAP2C-associated gene sets exhibited prominent up-up concordance (Figure 2A). TFAP2C has been shown to regulate gene expression associated with luminal breast epithelial cells and estrogen receptor signaling [47,48], suggesting that prioritized compounds coordinately suppress both FOXA1- and TFAP2C-dependent transcriptional programs. In addition, gene sets associated with KDM3A and RARA showed significant down-down concordance across prioritized compounds. KDM3A encodes a histone H3K9 demethylase implicated in estrogen receptor-dependent transcription [49], while RARA encodes a nuclear receptor whose activity intersects with estrogen receptor signaling [50]. GATA3-associated gene sets showed significant down-down concordance across prioritized compounds, indicating that genes reduced after GATA3 knockdown were likewise reduced by drug treatment. Given that GATA3 is a central regulator of mammary luminal-cell differentiation and a hallmark factor in luminal breast cancer [51], this enrichment further supports that prioritized compounds influence coordinated luminal transcriptional programs beyond FOXA1 alone. The coordinated enrichment of multiple TF-associated gene sets indicates that the transcriptional effects of prioritized compounds are not limited to FOXA1-regulated transcriptional programs but extend to additional TF-linked gene expression signatures. Given that FOXA1 functions as a pioneer factor that establishes chromatin accessibility for estrogen receptor binding [41], ESR1-associated gene sets would be expected to show concordance among prioritized compounds; however, ESR1 concordance was weaker than anticipated (Figure 2A), which may reflect the requirement for ligand-free nature of the TF knockdown datasets from KnockTF 2.0 [52]. ESR1-mediated transcription is highly context dependent, requiring estrogen ligand binding, receptor activation, accessible chromatin, and cooperation with luminal breast cancer regulators such as FOXA1, and GATA3 [53,54]. These molecular cooperation mechanisms are not fully represented in isolated TF knockdown signatures, particularly when the perturbation data are generated without ligand stimulation or matched hormonal context. As a result, TF knockdown signatures may capture only part of the regulatory program controlled by ligand-dependent transcription factors, potentially reducing the accuracy of concordance scoring for pathways that depend on receptor activation or cooperative chromatin recruitment. This limitation may affect the robustness of drug prioritization for TFs whose activity is highly context-dependent, and it highlights the need for future datasets that include ligand status, combinatorial TF perturbations, and matched epigenomic and transcriptomic profiling.

**Figure 3:**
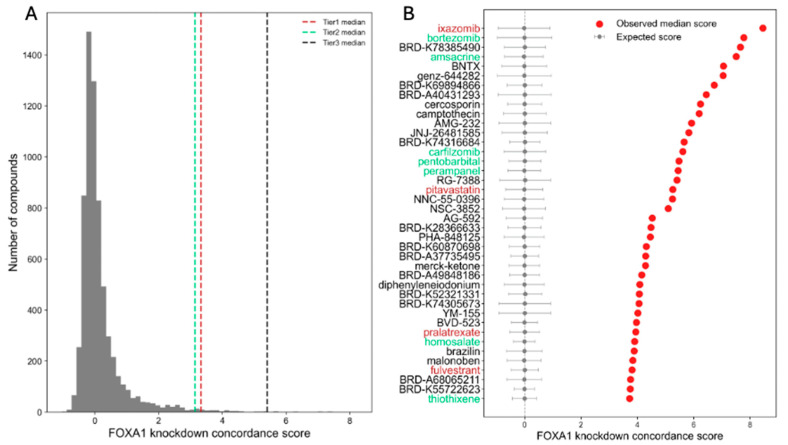
FOXA1 knockdown concordance scores quantify directional concordance between drug-induced and FOXA1 knockdown-induced gene expression. (**A**) Distribution of FOXA1 knockdown concordance scores across all 6605 compounds. Dotted vertical lines indicate median concordance scores for Tier 1 (red), Tier 2 (green), and Tier 3 (black) compounds. (**B**) Observed and expected FOXA1 knockdown concordance scores for the top 40 prioritized compounds. Compounds are ranked by the observed median FOXA1 knockdown concordance score across LINCS signatures in MCF7 cells (red points). Gray points and horizontal error bars indicate the permutation-derived expected score (null mean ± SD), estimated from random gene sets matched to the sizes of the FOXA1 knockdown-upregulated and knockdown-downregulated gene sets. Colored compound names indicate tier categories.

Among the 63 prioritized compounds, the FDA-approved selective estrogen receptor degrader (SERD) fulvestrant ranked fourth among Tier 1 compounds by FOXA1 knockdown concordance score, behind ixazomib, pitavastatin, and pralatrexate (Figure 3B). Fulvestrant degrades ESR1 protein at enhancers where FOXA1 establishes chromatin accessibility, thereby disrupting estrogen receptor-dependent transcription [39,55]. Its recovery among the top Tier 1 compounds is therefore expected: the transcriptional consequences of ESR1 degradation-suppression of estrogen receptor-dependent gene expression, are directionally concordant with FOXA1 knockdown-induced gene expression changes, as both converge on the same breast cancer transcriptional axis. The recovery of fulvestrant within the top Tier 1 compounds supports the biological validity of FOXA1-centered transcriptional concordance as a prioritization criterion. Among compounds with higher concordance scores, ixazomib and bortezomib-both proteasome inhibitors, ranked at the top of the candidate list (Figure 3B), highlighting the capacity of the framework to identify pharmacologically diverse mechanisms beyond the estrogen receptor axis.

To assess directional concordance between drug-induced transcriptional responses and FOXA1 suppression, we computed a FOXA1 knockdown concordance score for each compound across the full LINCS L1000 library treated in MCF7 (N = 6605 compounds; median = −0.02; IQR = 0.48; Figure 3A). The 63 prioritized compounds exhibited substantially higher concordance scores than the full LINCS background (median = 4.01; IQR = 1.84), with all three tiers significantly exceeding the background distribution (Tier 1: median = 3.94, *p* = 6.99 × 10^−5^; Tier 2: median = 3.73, *p* = 4.77 × 10^−10^; Tier 3: median = 4.06, *p* = 2.98 × 10^−30^; one-sided Mann-Whitney U test; Figure 3A). Across all 63 candidates combined, concordance scores were significantly elevated relative to the full compound library (*p* = 1.96 × 10^−41^; one-sided Mann-Whitney U test), suggesting that the selection criteria effectively enriched for compounds with transcriptional concordance with FOXA1 suppression. Concordance scores did not differ significantly across tiers (Tier 1 vs. Tier 2: *p* = 0.25; Tier 1 vs. Tier 3: *p* = 0.35; Tier 2 vs. Tier 3: *p* = 0.69; Mann-Whitney U test), consistent with the tier classification reflecting clinical evidence rather than transcriptional concordance strength. Permutation testing indicated that observed knockdown concordance scores of all 63 compounds significantly exceeded expected values which were the mean values of the empirical null distribution derived from 5000 random gene set permutations (*p* < 2.0 × 10^−4^ for all compounds; permutation null mean ranging from −0.024 to 0.021; one-sided permutation test; Figure 3B), demonstrating that directional concordance between drug-induced and FOXA1 knockdown-induced gene expression changes is statistically robust and consistent across prioritized candidates.

### 2.5. Variant-to-Gene Mapping Identifies Complementary Therapeutic Candidates

The TF-based prioritization strategy successfully identified 63 compounds through FOXA1 knockdown concordance. To complement this approach and identify additional candidates that may affect variant-linked genes through alternative regulatory mechanisms, we developed a parallel gene-based prioritization strategy that does not require TF perturbation data.

We mapped the 1537 candidate causal variants to genes based on proximity by linking two nearest genes within 1000 kb. This identified 347 genes as putative regulatory targets of breast cancer risk variants (Table S6). For each compound in the LINCS L1000 library, we tested whether the top 250 up-regulated or top 250 down-regulated genes showed significant enrichment for the 347 genes using Fisher’s exact test. Compounds with enrichment of *p* < 0.001 were retained as gene-based candidates. Analysis of down-regulated gene enrichment identified 53 compounds, including 15 approved drugs (28% approval rate, Table S7), representing 2.0-fold enrichment relative to the overall LINCS L1000 library baseline (odds ratio = 2.5, *p* = 0.004, one-sided Fisher’s exact test) (Figure 2B). This set included toremifene, a selective estrogen receptor modulator (SERM) approved for metastatic breast cancer treatment [56], and raloxifene, a SERM approved for breast cancer risk reduction in high-risk postmenopausal women [56]. Analysis of up-regulated gene enrichment identified 87 compounds, including 15 approved drugs (17% approval rate), though none with existing breast cancer indications (Table S8).

Comparison with the FOXA1-based candidate set revealed zero overlap between FOXA1-based (63 compounds) and gene-based set (53 down-enriched, 87 up-enriched). This lack of overlap is consistent with limited gene set intersection: FOXA1 knockdown affects 1780 genes, while variant mapping identified 347 genes, with only 32 genes (9%) in common. The three strategies collectively identified 203 unique candidate compounds, substantially expanding therapeutic coverage beyond any single analytical framework.

### 2.6. Prioritized Compounds Exhibit Transcriptional Signatures Anti-Correlated with Breast Cancer-Associated Pathways

**Figure 4:**
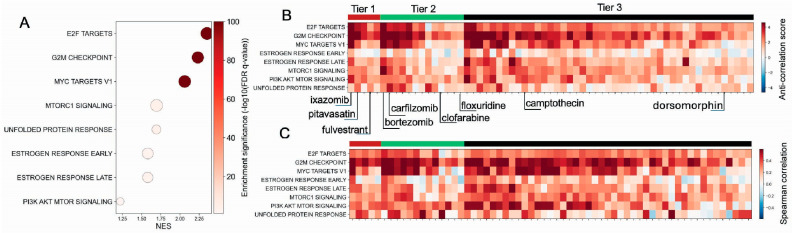
Prioritized compounds show transcriptional effects anti-correlated to core breast cancer-associated hallmark pathway activity. (**A**) Hallmark pathway enrichment in TCGA-BRCA differentially expressed genes (primary tumors versus matched normal breast tissue; DESeq2, FDR adjusted *p* < 0.01, |log_2_FC| > 2). NES, normalized enrichment score; positive values indicate enrichment among tumor-upregulated genes. (**B**) Pathway-level anti-correlation scores for 63 prioritized compounds across eight breast cancer-associated hallmark pathways. Color scale is centered at zero and capped at the 95th percentile of absolute values for visualization. (**C**) Rank-based directional concordance measured by Spearman correlation (ρ) between drug-induced gene expression changes and a signed disease direction vector (+1 for tumor-upregulated genes and −1 for tumor-downregulated genes within each pathway). Positive values indicate transcriptional changes anti-correlated to tumor-associated gene expression. Compound labels are colored by candidate tier. For readability, only representative compounds from each tier are labeled; the full compound list is provided in Supplementary Table S9.

Breast tumors are characterized by transcriptional programs associated with proliferation, cell-cycle progression, oncogenic growth signaling and hormone-responsive signaling [57]. In breast cancer, pathways such as E2F targets [58], G2M checkpoint [59], MYC targets [60], mTORC1 signaling [61], unfolded protein response [62], and estrogen response [63] have been associated with aggressive clinical features, metastasis risk, and endocrine-responsive biology. To evaluate whether the transcriptional effects of prioritized compounds are anti-correlated with disease-associated pathway activity, we defined breast cancer-associated transcriptional programs using differential gene expression analysis of primary tumors versus matched normal tissue, followed by hallmark pathway enrichment analysis (Figure 4A). These pathways were likewise among the canonical programs enriched in tumors.

To quantify the extent to which prioritized compounds are transcriptionally anti-correlated with breast cancer-associated pathway activity, we computed two complementary metrics for each of the 6605 LINCS compounds: an anti-correlation score, defined as the mean drug-induced z-score of tumor-downregulated pathway genes minus the mean z-score of tumor-upregulated pathway genes (positive values correspond to anti-correlated transcriptional changes to the tumor expression pattern), and a rank-based Spearman correlation between drug-induced z-scores and a signed disease direction vector (positive values indicating directional anti-correlation; see Section 4; Figure 4B,C). Among the 63 FOXA1-prioritized compounds, 34 (54%) exhibited positive anti-correlation scores across all eight evaluated hallmark pathways simultaneously, compared to 5.3% of the 6542 non-prioritized LINCS compounds (10.3-fold enrichment; *p* = 2.3 × 10^−26^, one-sided Fisher’s exact test). Consistent results were obtained using the Spearman metric (54% vs. 4.1% of non-prioritized compounds; 13.1-fold enrichment; *p* = 1.3 × 10^−29^), indicating that the enrichment is consistent across scoring approaches. An additional 23 compounds (37%) showed positive scores in seven of eight pathways, and the remaining six compounds (9%) in six of eight pathways, indicating that the majority of prioritized compounds exhibit transcriptional changes anti-correlated to core breast cancer pathway activity.

Cell proliferation-associated pathways showed the most consistent anti-correlated transcriptional changes across compounds: all 63 compounds exhibited positive scores for the G2M checkpoint (100%), 62 for E2F targets (98.4%), and 59 for MYC targets (93.6%; Figure 4B). Pathways related to growth and stress responses also showed similar patterns-mTORC1 signaling in 62 compounds (98.4%), PI3K/AKT/mTOR signaling in 60 (95.2%), and the unfolded protein response in 57 (90.5%). For hormone-responsive programs, 62 compounds (98.4%) showed positive scores for the estrogen response late signature, whereas a smaller subset (44 of 63; 69.8%) showed positive scores for the estrogen response early signature. This difference indicates that anti-correlated transcriptional changes are more consistently observed for late estrogen response genes than for early response genes [39]. Comparable results were obtained using the Spearman metric across all pathways (Figure 4C, Table S10), indicating consistent patterns across scoring approaches.

Collectively, 57 of 63 prioritized compounds (91%) showed positive scores in at least seven of eight core breast cancer hallmark pathways, indicating that FOXA1-centered prioritization is associated with compounds whose transcriptional effects anti-correlated with multiple disease-associated pathway signatures, supporting their prioritization for further evaluation.

Pathway anti-correlation analysis was also performed for gene-based candidates. Among 53 down-enriched gene-based candidates, 17 compounds (32%) showed positive anti-correlation across ≥ 6/8 pathways, though with more variable anti-correlated patterns compared to the FOXA1-based candidates (Figure S3). This variability likely reflects the diverse molecular mechanisms by which gene-based prioritized drugs may affect regulatory targets, in contrast to the more uniform transcriptional effects of compounds concordant with FOXA1 knockdown. Notably, toremifene and raloxifene showed strong anti-correlation of late estrogen response pathways, consistent with their SERM mechanism of action. Among 87 up-enriched gene-based candidates, 29 compounds (33%) showed positive anti-correlation across ≥ 6/8 pathways, with similar heterogeneity (Figure S4).

The coherent pathway-level effects observed for FOXA1-based candidates, compared to more variable patterns for gene-based prioritized candidates, suggest that TF-centered prioritization yields compounds with more focused transcriptional mechanisms, whereas gene-based prioritization captures broader regulatory diversity. Both strategies successfully identify compounds that engage disease-relevant pathways. FOXA1-based candidates show systematic anti-correlated effects by mimicking FOXA1 knockdown, while gene-based candidates provide mechanistic diversity by targeting variant-linked genes through multiple regulatory mechanisms.

### 2.7. Curated Drug-Gene Interactions Provide Independent Support for Prioritized Candidates

**Figure 5:**
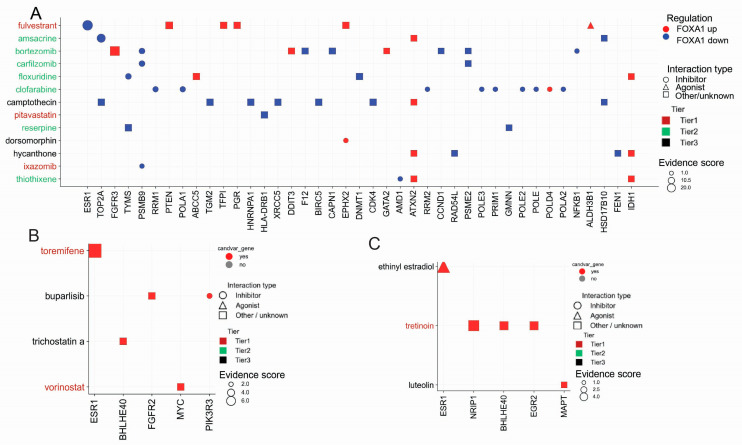
Drug-gene interactions for: (**A**) TF-centered approach prioritized compounds and FOXA1 knockdown-associated genes. Bubble plot showing documented drug-gene interactions from DGIdb 5.0 for 13 of 63 prioritized compounds with at least one interaction partner among genes differentially expressed upon FOXA1 knockdown in MCF7 cells (KnockTF 2.0; FDR adjusted *p* < 0.05). Each marker represents an interaction between a compound (y-axis) and a FOXA1 knockdown-regulated gene (x-axis). (**B**) Compound prioritized by down regulated genes and candidate variant mapped genes; (**C**) Compound prioritized by up regulated genes and candidate variant mapped genes. Marker size corresponds to the DGIdb evidence score supporting the interaction, marker shape indicates the annotated interaction type, and marker color indicates direction of FOXA1 regulation (upregulated or downregulated upon knockdown). Compound names are colored by candidate tier.

To determine whether the known molecular targets of prioritized compounds overlap with genes differentially expressed upon FOXA1 knockdown in MCF7 or candidate variant mapped genes, we cross-referenced each compound against DGIdb 5.0 [34], a publicly accessible database that aggregates drug-gene interaction data from multiple curated sources including ChEMBL [44], PharmGKB [64], and CIViC [65], and catalogs inhibitory, activating, and other experimentally supported interaction types between drugs and their molecular targets [34]. We then assessed whether documented drug targets overlapped with the genes differentially expressed upon FOXA1 knockdown in MCF7 (FDR adjusted *p* < 0.05 from differential expression analysis, as reported in KnockTF 2.0) or genes associated with the candidate variant predicted by the model. Among the 63 prioritized compounds prioritized by the FOXA1 approach, interactions between 13 (21%) compounds and genes differentially expressed upon FOXA1 knockdown were previously documented in DGIdb 5.0 [34], spanning Tier 1, Tier 2, and Tier 3 candidates (Figure 5A). These interactions provide independent support-derived from curated drug-target databases rather than transcriptional concordance scoring for the association between prioritized compounds and FOXA1-regulated gene expression.

Among Tier 1 compounds, fulvestrant has documented interactions with ESR1, PGR, PTEN, and ALDH3B1 in DGIdb 5.0 [34]. ESR1, for which fulvestrant has a documented inhibitory interaction, is among the genes downregulated upon FOXA1 knockdown in MCF7, indicating that fulvestrant’s known target is represented within the FOXA1-regulated gene set. Whether this overlap reflects direct ESR1 inhibition by fulvestrant, indirect effects mediated through FOXA1-dependent regulatory activity, or a combination of both cannot be determined from the present computational analysis.

Among Tier 2 compounds, the proteasome inhibitors bortezomib, carfilzomib, and ixazomib have documented interactions with PSMB9 in DGIdb 5.0 [34]; PSMB9 is among the genes downregulated upon FOXA1 knockdown in MCF7, indicating that these compounds share a documented target gene with FOXA1-regulated gene expression, though the functional significance of this overlap requires experimental validation. Pitavastatin has a documented interaction with HLA-DRB1 [66,67], which is also among genes differentially expressed upon FOXA1 knockdown in MCF7, consistent with a potential link between its immunomodulatory effects and FOXA1-regulated transcriptional changes. Clofarabine and floxuridine have documented interactions with multiple genes differentially expressed upon FOXA1 knockdown. For example, clofarabine interacts with RRM1, RRM2, and POLA1-DNA replication-associated genes downregulated upon FOXA1 knockdown-indicating overlap between its known targets and FOXA1-regulated gene expression. Although some individual drug-gene interactions exhibit directionality discordant with the FOXA1 knockdown-associated expression changes, the mechanistic basis of such discordance cannot be determined from curated interaction data alone and may reflect cell type-specific, condition-specific, or database annotation differences [34].

Among Tier 3 compounds, camptothecin and dorsomorphin have documented interactions with genes differentially expressed upon FOXA1 knockdown in MCF7; however, the interaction type for these compounds is not specified in the DGIdb 5.0, limiting interpretation of interaction directionality. Drug-gene interaction data were not used as a quantitative selection criterion but were incorporated as an independent qualitative filter, derived from curated drug-target databases rather than transcriptional data, alongside transcriptional concordance scoring and pathway-level anti-correlation analysis in the final compound shortlisting.

For compounds prioritized by gene-based approach, we tested for interactions between prioritized drugs and the 347 variant-linked genes. Among 53 down-regulated gene set enriched candidates, 4 compounds (8%) showed documented interactions with variant-linked genes (Figure 5B), while among 87 up-regulated gene enriched candidates, 3 compounds (3%) showed interactions (Figure 5C). This lower interaction rate compared to FOXA1-based candidates (21% vs. 3–8%) reflects the smaller gene set size: the 347 variant-linked genes represent a more limited target space in curated drug-gene databases compared to the 1780 FOXA1-regulated genes, reducing the probability of documented interactions.

Among downregulated gene prioritized candidates, toremifene showed interactions with ESR1 (estrogen receptor alpha), which present in both the variant-linked and FOXA1-regulated gene sets; vorinostat (histone deacetylase inhibitor) showed interactions with MYC; while buparlisib showed inhibitory interactions with PIK3R3 and unknown interaction with FGFR2, and trichostatin A showed interactions with and BHLHE40, though interaction types were not annotated. Among upregulated gene prioritized candidates, ethinyl estradiol showed agonist interaction with ESR1, indicating a direction opposite to the therapeutic hypothesis and this is consistent with literature report that excess breast cancer risk was associated with current use of triphasic ethinyl estradiol combined with levonorgestrel formulation in a prospective study of young women [68]; tretinoin (all-trans retinoic acid) showed interactions with NRIP1, BHLHE40, and EGR2; and luteolin showed interactions with MAPT, though interaction types remained unspecified.

The presence of drug-gene interactions for gene-based candidates, despite the smaller target gene set and lower overall interaction rates, provides mechanistic context for a subset of prioritized compounds. Notably, the identification of toremifene through gene-based prioritization, with documented ESR1 interaction and approved breast cancer indication, provides support for this strategy alongside the recovery of fulvestrant through FOXA1-based prioritization.

Combining transcriptional concordance scoring, gene enrichment, pathway-level anti-correlation analysis, and curated drug-gene interaction with experimental or clinical evidence, we derived a shortlist of candidate compounds (Table 1). These compounds collectively show high FOXA1 knockdown concordance scores or their treatment-induced differentially expressed genes significantly enriched in candidate variant mapped genes, transcriptional effects that are anti-correlated with core breast cancer-associated hallmark pathways across scoring metrics, and literature reported evidence. As a proof-of-concept application in breast cancer, these results demonstrate that integrating allele-specific enhancer modeling, TF-centered gene expression analysis, and curated drug-target data can prioritize candidate compounds linked to noncoding genetic risk. This framework can be extended to other complex traits and cellular contexts as matched epigenomic, perturbation, and drug transcriptional datasets become available.

## 3. Discussion

In this study, we developed an integrative computational framework that links disease-associated noncoding variants to drug repurposing candidates through allele-specific enhancer modelling, TF-centered transcriptional perturbation, and drug-induced transcriptional responses. Using breast cancer as a proof-of-concept, we identified candidate regulatory variants enriched for FOXA1-associated motif features and prioritized compounds through two complementary strategies: a TF-based approach based on concordance with FOXA1 knockdown signatures and a gene-based approach based on enrichment of variant-proximal putative target genes among drug-induced differentially expressed genes. Both strategies recovered approved breast cancer therapeutics, including fulvestrant, toremifene, and raloxifene, providing supporting evidence that model-predicted regulatory variants capture disease-relevant biology. In addition, prioritized compounds showed anti-correlated transcriptional changes with core breast cancer-associated hallmark pathways, and integration of pathway anti-correlation, drug-gene interactions, and clinical or experimental evidence refined the candidate list to 15 high-priority repurposing candidates. Together, these findings support a computational framework for translating noncoding genetic risk into pharmacologically relevant therapeutic hypotheses through regulatory variant modeling.

A central biological finding is the convergence of breast cancer risk variants on FOXA1-associated motif features. FOXA1 is a pioneer TF that establishes accessible chromatin states and coordinates estrogen receptor-dependent transcriptional programs in breast cancer [41]. Aberrant FOXA1 activity has also been implicated in endocrine-resistant and metastatic ER-positive breast cancer [40,69]. In our analysis, risk alleles showed strong preferential enrichment of FOXA1-associated motif features among 32 evaluated TF motifs, and FOXA1 was strongly upregulated among risk allele-enriched TFs in TCGA-BRCA primary tumors. These findings support a model in which dispersed noncoding variants converge on lineage-defining enhancer-associated regulatory programs linked to breast cancer-relevant transcriptional changes.

Importantly, TF motifs identified through TF-MoDISco reflect motif features prioritized by the enhancer prediction model based on attribution scores, rather than direct evidence of differential TF binding in vivo between alleles [30]. Therefore, enrichment of a motif in one allele indicates a stronger model-predicted regulatory contribution within that sequence context but does not imply absence of binding capacity or functional engagement in the alternate allele. This distinction is particularly relevant for pioneer factors such as FOXA1, which can bind broadly across accessible chromatin landscapes. Accordingly, our interpretation emphasizes allele-dependent differences in model-predicted regulatory contribution rather than strict presence or absence of binding events.

Although FOXA1 emerged as the strongest convergent factor based on allele-specific motif enrichment, tumor gene expression, prior functional evidence, and availability of compatible perturbation data, enhancer activity is typically governed by cooperative binding of multiple TFs [70]. Therefore, FOXA1 should not be interpreted as acting in isolation, but rather as a tractable regulatory axis supported by both motif enrichment and perturbation data. Consistent with this view, downstream drug perturbation analyses revealed coordinated changes in gene expression associated with additional TF-regulated gene sets, including TFAP2C-, RARA-, and KDM3A-associated signatures. These TFs have established roles in estrogen receptor-dependent and breast cancer-associated gene expression [48,49,71], suggesting that prioritized compounds may modulate a broader co-regulatory transcriptional programs beyond FOXA1 alone.

Discordance between allele-specific motif enrichment and bulk tumor expression for some TFs, such as RXRG and BATF3, is also biologically plausible. Motif enrichment may reflect variant-driven changes in the context of a local sequence that alter the relative contribution of individual TF motifs to enhancer prediction, without necessarily requiring concordant changes in TF expression in tumor tissue. Accordingly, drug prioritization was guided by directional concordance with FOXA1 knockdown-induced gene expression changes, reflecting modulation of a central regulatory axis rather than inhibition of FOXA1 in isolation.

Rather than directly identifying compounds that bind or inhibit a single TF, our framework prioritizes drugs whose transcriptional effects are directionally concordant with TF knockdown-induced gene expression changes in the corresponding cell line. The recovery of fulvestrant among the top Tier 1 compounds is consistent with the established co-dependency of estrogen receptor transcriptional activity on FOXA1-mediated chromatin accessibility [39], although whether this reflects direct ESR1 inhibition or indirect effects through FOXA1-dependent regulation requires experimental investigation. Beyond established therapies, several prioritized compounds have independent experimental or clinical evidence supporting their relevance in breast cancer. For example, ixazomib has shown antitumor activity in fulvestrant-resistant, advanced breast cancer in clinical studies [72], pitavastatin has been reported to overcome chemoresistance in breast cancer models [73], and proteasome inhibitors such as bortezomib and carfilzomib have shown preclinical antitumor activity in breast cancer systems [74,75]. Together, these examples support the pharmacological relevance of FOXA1-centered transcriptional concordance scoring and highlight additional candidates, including pitavastatin, floxuridine and clofarabine, for further experimental evaluation.

Gene-based prioritization mapped the 1537 deep learning-predicted candidate variants to 347 putative target genes and tests drug enrichment independent of TF annotation. This strategy identified 140 compounds, including approved breast cancer drugs such as toremifene and raloxifene. The recovery of these known drugs through variant-gene proximity alone, without TF perturbation data, provides supporting evidence that model-predicted regulatory variants can nominate therapeutically relevant candidate genes. Together with the TF-based recovery of fulvestrant, these findings support the biological plausibility of using deep learning-based regulatory variant prediction to generate therapeutic hypotheses through complementary prioritization strategies.

However, a conceptual limitation of the gene-based strategy is that its initial enrichment step does not inherently encode the desired direction of therapeutic modulation. In contrast to the TF-based approach, which evaluates directional concordance with TF knockdown-induced transcriptional changes, the gene-based method prioritizes compounds based on enrichment of variant-proximal genes among drug-induced differentially expressed genes. As a result, compounds may be ranked because they perturb relevant genes, even if their pharmacological action is not therapeutically favorable. Ethinyl estradiol illustrates this limitation: although it is connected to ESR1-associated biology, its agonistic activity through estrogen receptor signaling may be inconsistent with the therapeutic premise in breast cancer. Therefore, gene-based candidates should be interpreted as hypothesis-generating. In the final prioritization, we considered the direction of gene expression change, pathway-level anti-correlation, drug mechanism of action, and disease-specific pharmacological context.

Methodologically, this work extends regulatory deep learning approaches beyond variant annotation toward drug repurposing. TREDNet was selected because enhancer variant-effect prediction is central to the proposed framework and because prior comparative analyses supported its performance for this task. Nevertheless, the MCF7 enhancer prediction performance indicates that regulatory variant-effect modeling remains imperfect, and future work should continue to benchmark TREDNet against emerging regulatory sequence models, including Enformer [76], AlphaGenome [77], and related architectures. By integrating enhancer-based allele-specific modeling, TF knockdown-induced gene expression profiles, directional transcriptional concordance scoring, and pathway-level anti-correlation analysis, the framework incorporates upstream regulatory context into compound prioritization. This stepwise integration provides a quantitative strategy for translating noncoding genetic architecture into computationally supported therapeutic hypotheses.

The framework is designed to be extensible to additional disease-cell line pairs when matched epigenomic annotations, TF perturbation signatures, and drug-induced transcriptional datasets are available. In the present study, the complete drug prioritization workflow was demonstrated in MCF7 breast cancer as a proof-of-concept, whereas the upstream enhancer modeling and TF identification steps were explored across additional cell lines. Among 50 cell lines with overlapping LINCS and KnockTF coverage, seven retained sufficient epigenomic annotation after excluding cell lines with incomplete chromatin accessibility or H3K27ac profiles, or with documented prior perturbations such as chemical treatment or physical manipulation: A673, HCT116, HepG2, IMR90, K562, MCF7, and PC3. For each cell line, enhancers were defined by intersecting chromatin accessibility peaks with H3K27ac signals, yielding 29,000–98,000 cell type-specific enhancers per cell line. These enhancers were used to train cell type-specific enhancer models and to construct annotations for heritability enrichment analysis. TREDNet-based enhancer prediction models [22] achieved robust performance on chromosome-level held-out test sets, with AUROC values of 0.91–0.98 and AUPRC values of 0.58–0.86 across the seven cell lines (Figure S1A,B). We then applied LDSC partitioned heritability analysis to 135 GWAS summary statistics representing 42 traits selected based on their relevance to the seven cell lines analyzed. Significant heritability enrichment was observed for trait-cell line pairs consistent with known cell-type identity. In addition, representative TFs were identified in several cell line-disease pair contexts, including HNF4A in HepG2 for type 2 diabetes, ATF3 in HCT116 for colorectal cancer, JUND in PC3 for prostate cancer, KLF3 in K562 for blood-related traits, and FOXA1 in MCF7 for breast cancer (Table S11). These findings support the feasibility of extending the upstream components of the framework beyond MCF7, although full drug prioritization in additional disease contexts will require disease-specific biological interpretation, pharmacological evaluation, and validation.

Several limitations warrant consideration. First, inference of candidate regulatory variants and compound prioritization are computational and require further validation. Although the eQTL and FOXA1 ChIP-seq enrichment analyses provide independent functional support for the prioritized variants, they do not replace direct allele-specific validation using approaches such as MPRA, CRISPR-based enhancer perturbation, or allele-specific chromatin accessibility assays. Similarly, recovery of approved breast cancer drugs and pathway-level anti-correlation provide supportive computational evidence but do not establish independent validation of drug efficacy, particularly because drug prioritization and pathway anti-correlation both rely on LINCS drug-induced transcriptional profiles. Second, proximity-based variant-to-gene assignment does not fully capture long-range enhancer-promoter interactions or cell-type-specific chromatin architecture; future implementations should incorporate Activity-by-Contact models, promoter-capture Hi-C, eQTL mapping, and enhancer-gene interaction databases to improve regulatory variant-to-gene assignment. Third, applicability of the framework is constrained by the availability and overlap of epigenomic, TF perturbation, and drug-induced transcriptional datasets, limiting analyzable cellular contexts. In particular, although multiple enriched TF-associated motifs were identified, downstream drug prioritization required compatible TF perturbation signatures, which were unavailable or insufficient for most enriched motifs in the current framework. Therefore, the current study focused on FOXA1 as a tractable regulatory axis and did not explicitly model motif co-occurrence or combinatorial TF interactions. Fourth, drug-gene interaction databases (e.g., DGIdb 5.0 [34]) are incomplete and not cell type-specific, which may obscure context-dependent regulatory relationships. Finally, the use of cancer-derived cell lines introduces somatic alterations that may not fully reflect germline regulatory mechanisms. Enhancer prediction models also rely on available epigenomic annotations and may not fully capture regulatory dynamics under disease-specific conditions. These models predict enhancer probability rather than direct enhancer activity, and allele-specific differences in prediction scores do not constitute direct evidence of differential regulatory function in vivo.

Future work should prioritize independent validation and broader evaluation of the framework. This includes incorporating independent pharmacogenomic datasets, benchmarking against established drug-repurposing approaches, and experimentally testing prioritized compounds in relevant breast cancer models. In parallel, direct validation of prioritized regulatory variants using MPRA, CRISPR-based enhancer perturbation, or allele-specific chromatin accessibility assays will be important for confirming their regulatory effects. Expanding TF perturbation and drug transcriptional datasets across diverse cellular systems will further support application of enhancer-informed regulatory modeling to additional disease contexts, including non-cancer complex traits. Extending this framework to genetically driven neurological, immunological, and metabolic disorders may enable systematic identification of context-specific regulatory vulnerabilities. More broadly, these findings support the potential of enhancer-centered regulatory modeling as a foundation for translating noncoding genetic risk into computationally informed therapeutic hypotheses, pending validation across additional traits and cellular contexts.

## 4. Methods and Materials

### 4.1. Data Collection

#### 4.1.1. Drug-Induced Gene Expression Profiles

Phase I and II L1000 Gene Expression Profiles (978 Landmark Genes) Were Obtained From the Broad Institute LINCS Project [32] Via GEO (GSE70138 and GSE92742). Drug-Induced Gene Expression Z-Score Matrices Were Used for Downstream Analyses.

#### 4.1.2. TF Knockdown-Induced Gene Expression Profiles

TF Knockdown-Induced Gene Expression Profiles Were Retrieved From the KnockTF 2.0 Database [33], Comprising Perturbations of 456 TFs Across 357 Human Cell Lines. Up- and Down-Regulated Gene Sets (Adjusted *p* < 0.05 From Differential Expression Analysis, as Reported in the Database KnockTF 2.0) Were Extracted for Each TF-Cell Line Pair.

#### 4.1.3. Epigenomic Profiles

Chromatin Accessibility (DNase-Seq or ATAC-Seq) and Enhancer-Associated Histone Modification Profiles (H3K27ac) Were Obtained From ENCODE [28]. Fifty Cell Lines Overlapping Between LINCS and KnockTF Datasets Were Initially Considered. After Excluding Cell Lines with Incomplete Chromatin Accessibility or H3K27ac Profiles, or with Documented Prior Perturbations Including Chemical Treatment or Physical Manipulation Such as Ultrasonic Treatment, Seven Cell Lines Were Retained: A673, HCT116, HepG2, IMR90, K562, MCF7 and PC3.

#### 4.1.4. GWAS Summary Statistics

GWAS Summary Statistics for 135 Studies Spanning 42 Traits Were Downloaded From the GWAS Catalog. Traits Were Selected Based on Known Biological Association with the Cell Line of Origin (E.g., Breast Cancer Traits Paired with MCF7, Blood Traits Paired with K562). All Summary Statistics Were Harmonized to the GRCh38 Reference Genome.

### 4.2. Cell Type-Specific Enhancer Modeling

Enhancer prediction was performed using the TREDNet v0.1 two-phase deep learning framework [22]. Phase one was pretrained on 4560 genomic and epigenomic profiles from ENCODE v4 [29]. Phase two models were fine-tuned separately for each cell type using 2-kb genomic regions centered on open chromatin peaks overlapping H3K27ac signals. Coding regions, promoter-proximal regions (<2 kb from transcription start sites), and ENCODE blacklisted regions were excluded [78].

Each model was trained using fivefold cross-validation with chromosome-level holdout to prevent information leakage. Negative control sequences were sampled at a 10:1 ratio from all human open chromatin regions not overlapping positive enhancer sites or blacklisted regions.

### 4.3. GWAS Enrichment in Enhancer Regions

Linkage disequilibrium score regression (LDSC) (v1.0.1, python 3.0) [79] was used to evaluate enrichment of GWAS heritability within cell type-specific enhancer regions. Enhancer annotations were defined using 1-kb windows centered on predicted enhancer peak summits and annotated using the 1000 Genomes Project reference panel (hg38). GWAS summary statistics were processed in 500,000 SNP chunks using HapMap3 SNPs (w_hm3.snplist). Enrichment was assessed independently for each cell line. Statistical significance was determined from the regression coefficient estimated by LDSC using a z-test, with *p* < 0.05 considered significant.

### 4.4. Allele-Specific Enhancer Effect and Motif Inference

For breast cancer, which showed significant heritability enrichment within MCF7 enhancer annotations. For each variant, 2-kb sequences centered on the variant position were scored under both risk and protective alleles using the MCF7 enhancer model.

The allele-specific enhancer difference (Δscore) was defined as:

Δscore = P (risk allele) − P (protective allele)

where P represents the predicted probability of the cell type-specific enhancer model for the allele-centered 2-kb sequence. Absolute Δscore values were used to quantify allele-specific differences in model-predicted enhancer probability. Candidate regulatory variants were defined using two criteria: (i) at least one allele exceeded the model-derived enhancer probability threshold corresponding to a false positive rate of 10%, and (ii) the absolute allele-specific prediction difference (|Δscore|) ranked within the top 10% of all evaluated GWAS variants (above the 90th percentile of the empirical |Δscore| distribution). The FPR < 10% enhancer probability threshold was selected to prioritize high-confidence enhancer predictions while maintaining sufficient sensitivity for downstream analyses and has been used in a previous application of the same enhancer prediction framework for regulatory variant prioritization [23]. The top 10% |Δscore| threshold was selected to prioritize variants with strong predicted allele-specific regulatory effects while retaining a sufficient number of sequences for downstream DeepLIFT (v0.6.13.0) and TF-MoDISco (v2) motif discovery.

To assess functional support for the candidate regulatory variants, we tested whether they were enriched in independent functional genomics datasets, including breast cancer eQTLs from *Li et al.* [38] and MCF7 FOXA1 ChIP-seq peaks from ENCODE, using Fisher’s exact test.

Attribution scores were computed using DeepLIFT [30,80] with the negative enhancer sequence as background. TF-MoDISco [31] was applied to 500-bp windows centered on prioritized variants, importance-weighted subsequences, termed seqlets, representing recurrent sequence patterns within attribution score profiles (maximum 2000 per metacluster).

### 4.5. TF Enrichment in Drug Perturbation Profiles

For each TF-cell line pair with significant perturbation effects in KnockTF (adjusted *p* < 0.05 from differential expression analysis, as reported in the database KnockTF 2.0), up- and down-regulated gene sets were extracted. For each drug-cell line perturbation in LINCS, the top 250 up- and down-regulated genes were selected based on the z-score, consistent with standard LINCS L1000 analysis approaches [32].

Overlap between drug-induced gene sets and TF knockdown gene sets was assessed using Fisher’s exact test separately for up regulated genes upon TF knockdown in drug-induced upregulated genes (up-up) and down regulated genes upon TF knockdown in drug-induced downregulated genes (down-down) comparisons. TF enrichment *p*-values were computed for each drug-TF pair. For drugs with multiple perturbation profiles, the median enrichment *p* value was used.

### 4.6. TF Knockdown Concordance Score

To quantify directional concordance between drug-induced and TF knockdown-associated transcriptional responses, a TF knockdown concordance score was calculated as:

KD _concordance_ = Mean (z-up) − Mean (z-down)

where Mean (z-up) and Mean (z-down) represent the average drug-induced z-scores for genes up-regulated and down-regulated in the TF knockdown-induced differentially expressed gene set, respectively. This formulation follows the connectivity score principle of the connectivity map framework [81], adapted to quantify directional concordance with TF knockdown-induced gene expression changes. For drugs with multiple perturbation profiles, the median concordance score across experiments was used. Statistical significance of the KD concordance score was assessed using an empirical permutation test that preserves the sizes of the TF knockdown up- and down-regulated gene sets. For each drug-TF pair, random gene sets of equal sizes (number of up- and down-regulated genes) were sampled without replacement from the L1000 landmark gene universe, and the concordance score was recomputed across 5000 permutations to generate a null distribution. *p*-values were adjusted for multiple testing using the Benjamini-Hochberg FDR procedure.

Concordance score distributions were compared between prioritized compound tiers and the full LINCS background using one-sided Mann-Whitney U tests. Pairwise comparisons between tiers were performed using two-sided Mann-Whitney U tests.

### 4.7. Drug Prioritization

#### 4.7.1. TF-Based Prioritization

Candidate Compounds Were Prioritized by Requiring Statistically Significant Directional Enrichment in Both Up-Up and Down-Down Comparisons (Fisher’s Exact Test, *p* < 0.05) and A TF Knockdown Concordance Score Within the Top 10th Percentile Across All Evaluated LINCS Compounds. The 10th Percentile Threshold Was Selected Empirically Based on Maximization of Approved Drug Enrichment Across Threshold Values Spanning the 75th to 95th Percentile.

#### 4.7.2. Gene-Based Prioritization

Candidate Regulatory Variants Identified From the Enhancer Model Were Assigned to Putative Target Genes Using A Reproducible Proximity-Based Annotation Strategy. Specifically, Each Variant Was Mapped to the Two Nearest Genes Within A Maximum Distance of 1000 Kb. This Approach Was Used as A First-Pass Annotation for Enrichment-Based Drug Prioritization Rather Than as Evidence of Experimentally Confirmed Enhancer-Gene Interactions. Candidate Compounds Were Then Prioritized Based on Enrichment of Variant-Mapped Genes Among the Top 250 Up- or Down-Regulated Genes Induced by Drug Treatment in the Lincs L1000 Dataset, Using A One-Sided Fisher’s Exact Test with A Significance Threshold of *p* < 0.001.

### 4.8. Pathway-Level Anti-Correlation Analysis

For the breast cancer proof-of-concept analysis, differentially expressed genes (DEGs) were derived from TCGA-BRCA tumor versus matched normal RNA-seq data using TCGAbiolinks in R (v4.5) [82], with differential expression performed using DESeq2 (v1.50, FDR adjusted *p* < 0.01, |log2 FC| > 2, Wald test). Hallmark pathway enrichment was assessed using preranked GSEA (v4.4) [83]. For each drug and pathway, we computed two complementary anti-correlation statistics: a raw score defined as the mean drug-induced z-score of disease-downregulated genes minus mean z-score of disease-upregulated genes, and a rank-based directional concordance score defined as the Spearman correlation between rank-transformed drug-induced z-scores and a signed disease direction vector. Positive values indicate transcriptional changes anti-correlated to the disease-associated expression pattern. This formulation follows the principle of gene expression anti-correlation described in the connectivity map framework [81] and uses a rank-based correlation approach analogous to methods employed in gene set enrichment analysis [83]. To contextualize these effects among prioritized compounds, the same metrics were computed for all 6605 LINCS compounds treated in MCF7.

The drug prioritization and pathway anti-correlation analyses used LINCS drug-induced transcriptional signatures for compound-level responses but compared them against different biological reference signatures. TF-based prioritization evaluated concordance with FOXA1 knockdown-induced differentially expressed genes from KnockTF, whereas pathway anti-correlation evaluated whether drug-induced transcriptional changes were directionally opposite to TCGA-BRCA breast cancer-associated pathway signatures. Therefore, pathway anti-correlation was used as a complementary transcriptomic consistency assessment rather than as an independent validation.

### 4.9. Drug Annotation and Drug-Gene Interaction Analysis

Drug approval status and clinical trial annotations were obtained from DrugCentral [43] and ChEMBL [44] (accessed December 2025). Curated drug-gene interaction data were retrieved from DGIdb 5.0 [34] (accessed December 2025) and used as an independent source of drug-target evidence to assess overlap between documented drug targets and genes differentially expressed upon FOXA1 knockdown in MCF7.

## 5. Conclusions

In this study, we developed a regulatory variant-guided drug repurposing framework that connects noncoding genetic variation to candidate therapeutics through deep learning-based enhancer modeling. Using breast cancer as a proof-of-concept, we prioritized compounds through two complementary strategies: a TF-based approach based on FOXA1 knockdown concordance and a gene-based approach based on enrichment of variant-proximal putative target genes among drug-induced differentially expressed genes. The recovery of approved breast cancer therapeutics, including fulvestrant, toremifene, and raloxifene, supports the biological plausibility of the framework and demonstrates consistency with known breast cancer pharmacology. Integration of pathway anti-correlation, drug-gene interaction evidence, approval status, breast cancer trial information, mechanism of action, and literature support refined the broader candidate list to 15 shortlisted compounds with translational relevance. These included pharmacologically diverse agents such as proteasome inhibitors, epigenetic modulators, antimetabolites, topoisomerase-related agents, and metabolic or signaling modulators, with candidates such as ixazomib and pitavastatin supported by prior clinical or experimental evidence in breast cancer-related contexts. Overall, these findings suggest that modeling allele-specific enhancer effects can generate pharmacologically interpretable therapeutic hypotheses from noncoding genetic risk. The prioritized compounds should be considered computationally supported candidates for further evaluation, requiring validation in independent pharmacogenomic datasets and experimental breast cancer models.

## Figures and Tables

**Table 1:** Final prioritized drug candidates with mechanism of action and literature support.

Drug	Rank Method	Approve Any	BC Trial	MOA	Indication	Literature Support Evidence
Ixazomib	TF-FOXA1	1	1	proteasome inhibitor	multiple myeloma	Ixazomib in combination with fulvestrant demonstrated a favorable safety profile and antitumor activity in patients with fulvestrant-resistant, advanced estrogen receptor-positive breast cancer. NCT02993094; “https://doi.org/10.1002/onco.13733”
Pitavasatin		1	1	HMG-CoA reductase inhibitor	hypercholesterolemia and dyslipidemia	Pitavastatin was reported to overcome paclitaxel resistance in triple-negative breast cancer models. (NCT04705909; PMID: 41126317)
Bortezomib		1	0	proteasome inhibitor	multiple myeloma	Bortezomib has been shown to inhibit breast cancer growth in preclinical studies. (PMID: 20843837)
Carfilzomib		1	0	proteasome inhibitor	multiple myeloma	An albumin-coated nanocarrier formulation of carfilzomib demonstrated improved metabolic stability and enhanced cytotoxic effects in breast cancer cells in vitro. (PMID: 30954620)
Floxuridine		1	0	antimetabolite	liver metastases of gastrointestinal malignancy	Camptothecin-floxuridine conjugate nanocapsules enhanced anti-metastatic efficacy in breast cancer models. “https://pubs.acs.org/doi/10.1021/acsami.8b11723 (accessed on 15 January 2026)”
Clofarabine		1	0	inhibitor of ribonucleotide reductase	leukemias	Clofarabine exhibits potent anti-breast cancer activity, although cytotoxicity toward normal cells has been reported; modified analogs have shown improved selectivity. “https://doi.org/10.1016/j.bmcl.2025.130349”
Camptothecin		0	0	topoisomerase inhibitor		Tumor-targeted nanocrystal formulations of camptothecin demonstrated improved therapeutic efficacy in breast cancer models. “https://doi.org/10.1016/j.xphs.2025.103951”
Dorsomorphin		0	0	selective and ATP-competitive AMPK inhibitor		Dorsomorphin has been reported as an inhibitor of dickkopf-1 (DKK1), with implications for breast cancer progression. (PMID: 32001001)
Vorinostat	Gene down	1	1	histone deacetylase inhibitor (HDI)	cutaneous T-cell lymphoma	In combination with other conventional chemotherapeutics, exhibit anti-neoplastic properties through inhibition of proliferation, migration and invasion, induction of differentiation and apoptosis as well as cell-cycle arrest, in many types of BC cells, both in in vitro and in vivo settings. (PMID: 34572928)
Dicoumarol		1	0	anticoagulant agent, inhibits vitamin K reductase	deep vein thrombosis	In estrogen receptor-negative breast cancer (in vitro and in vivo), dicoumarol counteracted the chemoresistance to taxane-anthracycline-based chemotherapy by targeting the PSG1. (PMID: 27653744)
U-0126		0	0	MEK1/2 inhibitor		MEK inhibitor U-0126 reduces cancer cell proliferation and can induce cell death (apoptosis) in various breast cancer cell lines. (PMID: 22945392)
Trichostatin A (BRD-A19037878)		0	0	histone deacetylase inhibitor		Trichostatin A reverses epithelial-mesenchymal transition and attenuates invasion and migration in MCF-7 breast cancer cells. (PMID: 32104221)
Cyproheptadine	Gene up	1	0	combined serotonin and histamine antagonist	allergic symptoms	Treatment of human breast cancer cells (MCF7 cells) with cyproheptadine decreased the expression and transcriptional activity of ERα, thereby inhibiting estrogen-dependent cell growth. (PMID: 27088648)
Duloxetine		1	0	Selective Serotonin and Norepinephrine Reuptake Inhibitor (SNRI)	depression and anxiety	Treatment of human breast cancer cells (MCF7 cells) with cyproheptadine decreased the expression and transcriptional activity of ERα, thereby inhibiting estrogen-dependent cell growth. (PMID: 27088648)
Mitoxantrone		1	0	DNA-reactive agent, a potent inhibitor of topoisomerase II	prostate cancer; Acute myeloid leukemia; multiple sclerosis	mitoxantrone binds to eEF-2K and inhibits its activity, and the combination treatment of mitoxantrone and mTOR inhibitor resulted in significant synergistic cytotoxicity in breast cancer. “https://www.nature.com/articles/s41419-020-03153-x (accessed on 20 May 2026)”
Fluphenazine		1	0	blocking postsynaptic dopaminergic D1 and D2 receptors	schizophrenia and other psychotic disorders	Flu inhibited survival of metastatic TNBC cells. (PMID: 30949404)

## Data Availability

The data and resources used in this study are publicly available as follows: the pre-trained TREDNet model has been deposited at https://doi.org/10.5281/zenodo.8161621. Heritability partitioning analyses were performed using the standard S-LDSC implementation available at (https://github.com/bulik/ldsc, accessed on 14 June 2026). Human population variant data for heritability partitioning were sourced from the 1000 Genomes Project Phase 3, while baseline-LD model annotations (v2.2) were obtained from “https://doi.org/10.5281/zenodo.10515792”. Breast cancer GWAS summary statistics were downloaded from the GWAS Catalog (accession GCST004988) at “https://www.ebi.ac.uk/gwas (accessed on 23 June 2025)”. Drug-induced gene expression profiles were obtained from GEO database (GSE70138; GSE92742); Epigenomic profiles were downloaded from ENCODE; knockTF dataset was downloaded from “http://www.licpathway.net/KnockTF/index.html (accessed on 21 May 2025)”.

## Supplementary Materials

The following supporting information can be downloaded at: https://www.mdpi.com/article/10.3390/ph19071097/s1, Figure S1: Cell line-specific enhancer model performance and GWAS heritability enrichment. (A) Enhancer model performance (AUC) on the test set from a representative fold of chromosome-level cross-validation. (B) Enhancer model performance (AUPRC) on the test set from a representative fold of chromosome-level cross-validation. (C) GWAS heritability enrichment across cell line-specific enhancer annotations. Figure S2: Allele-dependent motif enrichment and tumor expression concordance in breast cancer. (A) Transcription factor motifs identified from breast cancer GWAS variants using TREDNet enhancer models and attribution-based TF-MoDISco analysis, ranked by log_2_ risk/protective enrichment. Dot size indicates seqlet support; color represents −log_10_(q-value). (B) Motif enrichment (x-axis; log_2_ risk/protective ratio) versus tumor differential expression in TCGA-BRCA (y-axis; log_2_ fold change). Concordant TFs (green) show agreement between risk allele enrichment and tumor upregulation. Point size reflects −log_10_(FDR). Dashed lines indicate classification thresholds. (C) Distribution of absolute allele-dependent enhancer prediction differences (|Δscore|) across all variants (gray) and prioritized candidates (red). The dashed vertical line marks the 90th percentile cutoff. Motif enrichment values were computed as log_2_((n_risk + 0.5)/(n_protect + 0.5)), applying a pseudocount of 0.5 (Haldane-Anscombe correction) to avoid undefined values when the protective allele seqlet count was zero. Figure S3: Prioritized compounds based on the enrichment of variant mapped genes in the downregulated genes under compound treatment show transcriptional effects anti-correlated to core breast cancer-associated hallmark pathway activity. (A) Pathway-level anti-correlation scores for 53 prioritized compounds across eight breast cancer-associated hallmark pathways. Color scale is centered at zero and capped at the 95th percentile of absolute values for visualization. (B) Rank-based directional concordance measured by Spearman correlation (ρ) between drug-induced gene expression changes and a signed disease direction vector (+1 for tumor-upregulated genes and −1 for tumor-downregulated genes within each pathway). Positive values indicate transcriptional changes anti-correlated to tumor-associated gene expression. Compound labels are colored by candidate tier. Figure S4: Prioritized compounds based on the enrichment of variant mapped genes in the upregulated genes under compound treatment show transcriptional effects anti-correlated to core breast cancer-associated hallmark pathway activity. (A) Pathway-level anti-correlation scores for 87 prioritized compounds across eight breast cancer-associated hallmark pathways. Color scale is centered at zero and capped at the 95th percentile of absolute values for visualization. (B) Rank-based directional concordance measured by Spearman correlation (ρ) between drug-induced gene expression changes and a signed disease direction vector (+1 for tumor-upregulated genes and −1 for tumor-downregulated genes within each pathway). Positive values indicate transcriptional changes anti-correlated to tumor-associated gene expression. Compound labels are colored by candidate tier. Table S1: Enrichment of candidate regulatory variants in breast cancer eQTL and MCF7 FOXA1 ChIP-seq peaks. Table S2: Sensitivity analysis of candidate regulatory variant enrichment across alternative |Δscore| thresholds. Table S3: Summary of TF motif enrichment and tumor expression. Log_2_ enrichment values were computed as log_2_((n_risk + 0.5)/(n_protect + 0.5)) using a pseudocount of 0.5 (Haldane-Anscombe correction). Negative log_10_(q-values) are reported for statistical significance. Table S4: Enrichment of approved drugs among candidate compounds at varying thresholds. *p*-values were calculated using Fisher’s exact test. Table S5: Candidate compounds prioritized by FOXA1-based approach. Table S6: Candidate regulatory variants mapped genes. Table S7: Candidate compounds prioritized by gene enrichment from the downregulated genes. Table S8: Candidate compounds prioritized by gene enrichment from the upregulated genes. Table S9: Compound names corresponding to the column order shown from left to right in Figure 4B,C. Table S10: Number and fraction of candidate compounds with positive pathway anti-correlation scores and Spearman correlation (spr) across pathways. Table S11: Overlap TF motif between TFmodisco identified and knockTF in each cell line-trait pair.
