# Supplementary material for: Using Deep Learning Models of Gene Regulation to Guide Drug Prioritization
Source: Pharmaceuticals (Basel). 2026 Jul 16;19(7):1097. doi: 10.3390/ph19071097 (PMC13414701; doi:10.3390/ph19071097)
Supplement: Supplementary file 1 [file pharmaceuticals-19-01097-s001.zip › supplementary figures1-s4.pdf]

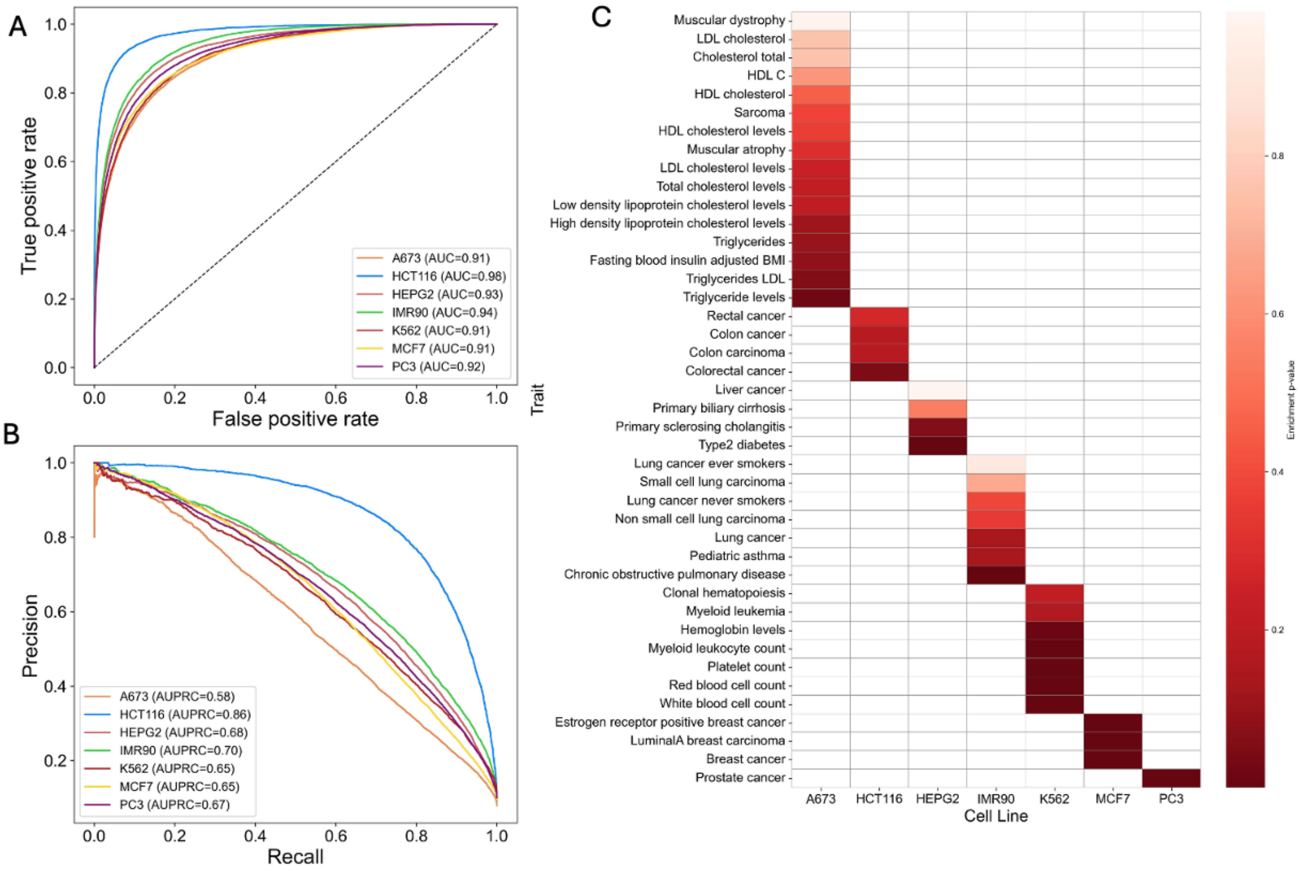

Figure S1 Cell line-specific enhancer model performance and GWAS heritability enrichment. (A) Enhancer model performance (AUC) on the test set from a representative fold of chromosome-level cross-validation. (B) Enhancer model performance (AUPRC) on the test set from a representative fold of chromosome-level cross-validation. (C) GWAS heritability enrichment across cell line-specific enhancer annotations.

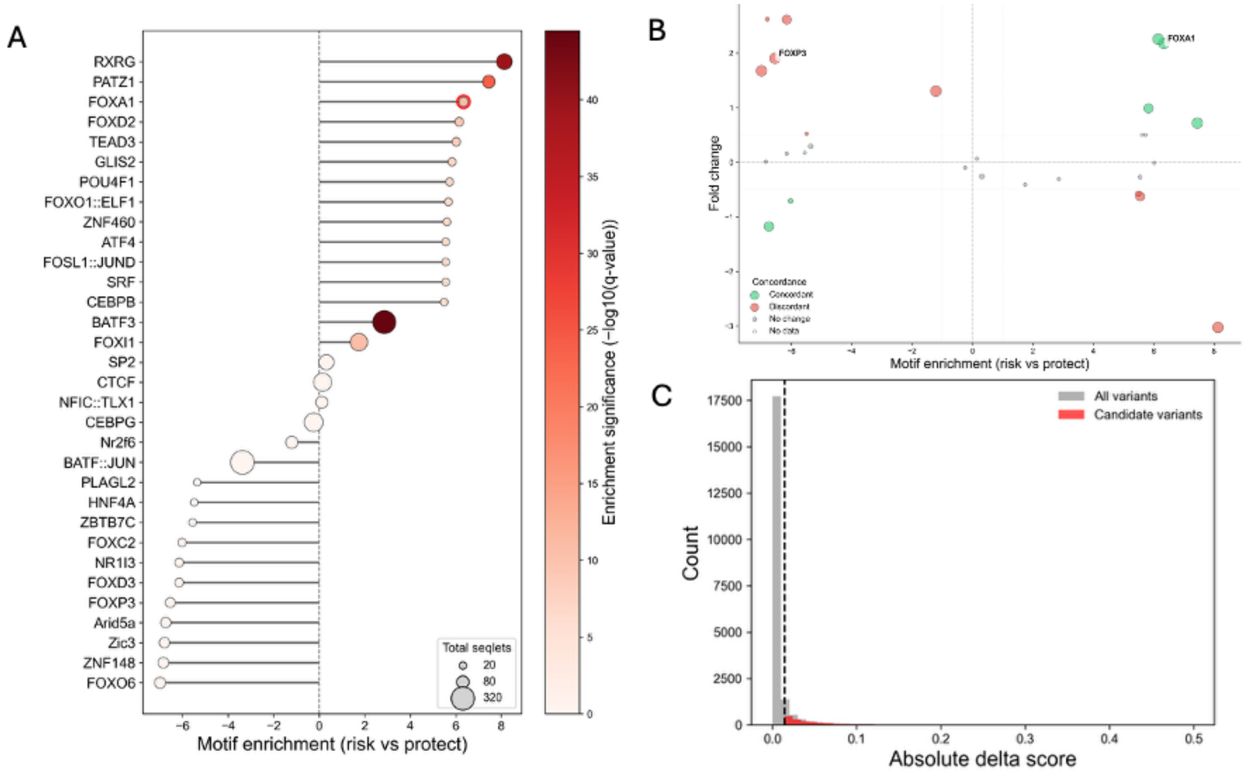

Figure S2. Allele-dependent motif enrichment and tumor expression concordance in breast cancer. (A) Transcription factor motifs identified from breast cancer GWAS variants using TREDNet enhancer models and attribution-based TF-MoDISco analysis, ranked by $\log_2$ risk/protective enrichment. Dot size indicates seqlet support; color represents $-\log_{10}$(q-value). (B) Motif enrichment (x-axis; $\log_2$ risk/protective ratio) versus tumor differential expression in TCGA-BRCA (y-axis; $\log_2$ fold change). Concordant TFs (green) show agreement between risk allele enrichment and tumor upregulation. FOXA1 and FOXD2 exhibit strong concordance. Point size reflects $-\log_{10}$(FDR). Dashed lines indicate classification thresholds. (C) Distribution of absolute allele-dependent enhancer prediction differences (|Δscore|) across all variants (gray) and prioritized candidates (red). The dashed vertical line marks the 90th percentile cutoff. Motif enrichment values were computed as $\log_2((n\_risk + 0.5)/(n\_protect + 0.5))$, applying a pseudocount of 0.5 (Haldane-Anscombe correction) to avoid undefined values when the protective allele seqlet count was zero.

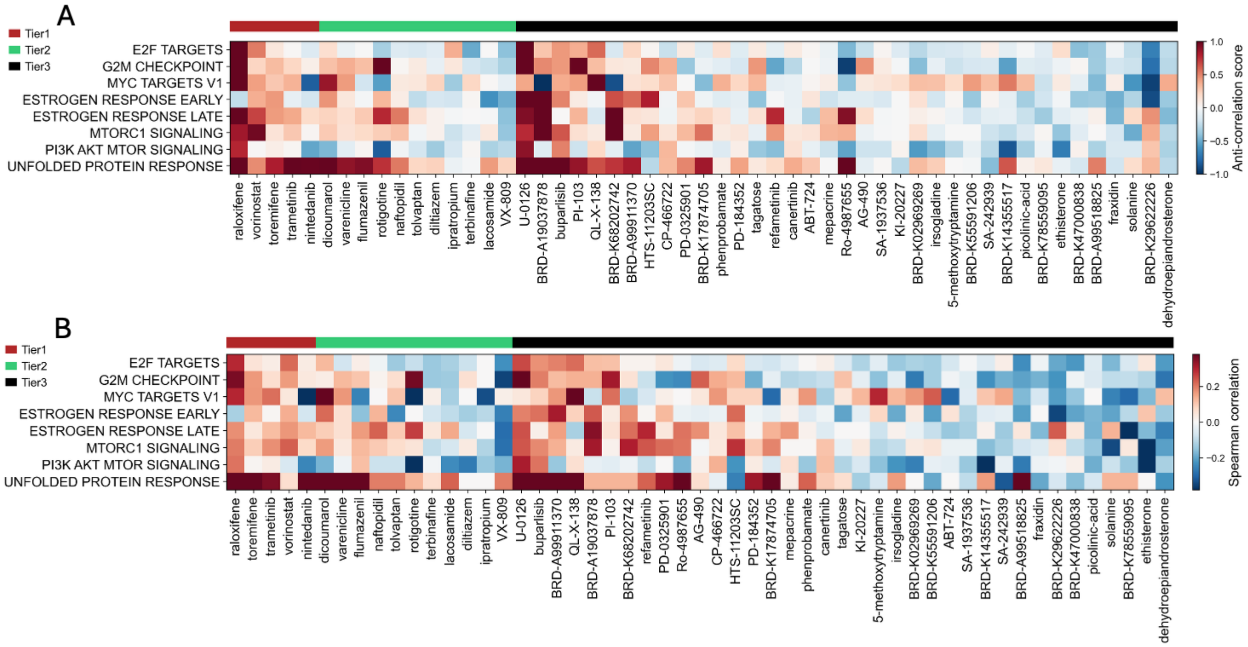

Figure S3. Prioritized compounds based on the enrichment of variant mapped genes in the downregulated genes under compound treatment show transcriptional effects anti-correlated to core breast cancer-associated hallmark pathway activity**.** (A) Pathway-level anti-correlation scores for 53 prioritized compounds across eight breast cancer-associated hallmark pathways. Color scale is centered at zero and capped at the 95th percentile of absolute values for visualization. (B) Rank-based directional concordance measured by Spearman correlation (ρ) between drug-induced gene expression changes and a signed disease direction vector (+1 for tumor-upregulated genes and -1 for tumor-downregulated genes within each pathway). Positive values indicate transcriptional changes anti-correlated to tumor-associated gene expression. Compound labels are colored by candidate tier.

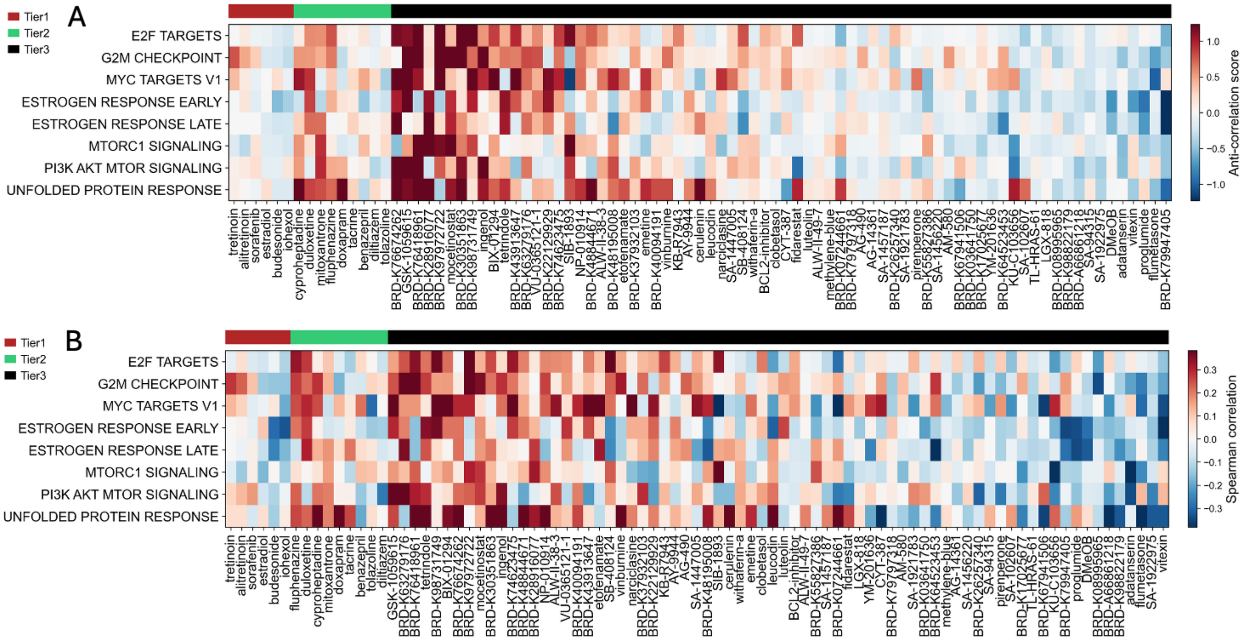

Figure S4. Prioritized compounds based on the enrichment of variant mapped genes in the upregulated genes under compound treatment show transcriptional effects anti-correlated to core breast cancer-associated hallmark pathway activity**.** (A) Pathway-level anti-correlation scores for 87 prioritized compounds across eight breast cancer-associated hallmark pathways. Color scale is centered at zero and capped at the 95th percentile of absolute values for visualization. (B) Rank-based directional concordance measured by Spearman correlation (ρ) between drug-induced gene expression changes and a signed disease direction vector (+1 for tumor-upregulated genes and -1 for tumor-downregulated genes within each pathway). Positive values indicate transcriptional changes anti-correlated to tumor-associated gene expression. Compound labels are colored by candidate tier.
